# Clinical impact of MMP and TIMP gene polymorphisms in gastric cancer

**DOI:** 10.1038/sj.bjc.6603307

**Published:** 2006-08-29

**Authors:** F J G M Kubben, C F M Sier, M J W Meijer, M van den Berg, J J van der Reijden, G Griffioen, C J H van de Velde, C B H W Lamers, H W Verspaget

**Affiliations:** 1 Department of Gastroenterology and Hepatology, Leiden University Medical Center, Leiden, The Netherlands; 2 Department of Oncologic Surgery, Leiden University Medical Center, Leiden, The Netherlands

**Keywords:** survival, Borrmann, Laurén, *Helicobacter pylori*, protein level

## Abstract

Gastric cancers express enhanced levels of matrix metalloproteinases (MMPs) and their tissue inhibitors (TIMPs). Single-nucleotide polymorphisms (SNPs) in MMP and TIMP genes may be associated with disease susceptibility and might also affect their antigen expression. We studied the genotype distribution and allele frequencies of SNPs of MMP-2, -7, -8 and -9 and TIMP-1 and -2 in gastric cancer patients in relation to tumour progression, patient survival and tissue antigen expression. The genotype distribution and allele frequencies were similar in gastric cancer patients and controls, except for MMP-7_−181A>G_. In addition, the genotype distribution of MMP-7_−181A>G_ was associated with *Helicobacter pylori* status (*χ*^2^ 7.8, *P*=0.005) and tumour-related survival of the patients. Single-nucleotide polymorphism TIMP-2_303C>T_ correlated significantly with the WHO classification (*χ*^2^ 5.9, *P*=0.03) and also strongly with tumour-related survival (log rank 11.74, *P*=0.0006). Single-nucleotide polymorphisms of MMP-2, -8, -9 and TIMP-1 were not associated with tumour-related survival. Only the gene promoter MMP-2_−1306C>T_ polymorphism correlated significantly with the protein level within the tumours. First-order dendrogram cluster analysis combined with Cox analysis identified the MMP-7_−181A>G_ and TIMP-2_303C>T_ polymorphism combination to have a major impact on patients survival outcome. We conclude that MMP-related SNPs, especially MMP-7_−181A>G_ and TIMP-2_303C>T_, may be helpful in identifying gastric cancer patients with a poor clinical outcome.

In the process of tumour dissemination and metastasis, matrix metalloproteinases (MMPs) and their tissue inhibitors (TIMPs) play an important role in the invasion of tissue, vascular and lymphatic basal membranes and the subsequent coordinated proteolytic breakdown and reconstitution of extracellular matrix (Kohn and Liotta, 1995). Matrix metalloproteinases also modulate cell proliferation, apoptosis and host immune surveillance (Egeblad and Werb, 2002). Immunohistochemical and *in situ* hybridisation studies as well as quantitative assays have demonstrated that gastric carcinomas contain enhanced amounts of MMPs (Nomura *et al*, 1995; Honda *et al*, 1996; Mori *et al*, 1997). We previously reported significantly enhanced MMP and TIMP levels in gastric carcinomas, but only MMP-2 was independently associated with a poor overall survival of the patients (Kubben *et al*, 2006). Single-nucleotide polymorphisms (SNPs) within MMP genes are thought to influence the expression of MMPs and/or even seem to be associated with the susceptibility for the development of malignancy. For instance, a functional SNP in the MMP-2 gene promoter (−1306C>T) was found to be associated with the risk of the development, but not the metastatic behaviour of gastric cardia adenocarcinoma, in an ethnic Chinese population (Miao *et al*, 2003). Furthermore, the frequency of a functional SNP of MMP-7 (−181A>G) was found to be significantly higher in gastric cardiac carcinoma patients compared to controls in another Chinese study (Zhang *et al*, 2005). Particularly, genotypes with the MMP-7_−181G_ allele (A/G+G/G) showed a significantly increased susceptibility for gastric cardiac carcinoma with an odds ratio of 1.96 (Zhang *et al*, 2005). Finally, a significant association in Japanese gastric cancer patients was found between an SNP in the promoter of the MMP-9 gene (−1562C>T) and the degree of tumour invasion, clinical stage and lymphatic invasion (Matsumura *et al*, 2005). However, as indicated above, these studies on MMP-SNPs in gastric carcinoma patients describe ethnic Chinese and Japanese populations with a known high incidence of gastric cancer.

In the present study, we determined the genotype distribution and allele frequencies of SNPs of MMP-2, -7, -8 and -9, and of TIMP-1 and -2 in a cohort of 79 Caucasian gastric carcinoma patients, in which we previously assessed clinical relevance of the respective protein levels. In order to get insight into the functional and clinical contribution of these MMP-related gene polymorphisms, we assessed the relation between the distribution of these SNPs and the respective protein levels in tumour and adjacent normal tissue as well as the relation of the SNPs with established clinico-pathological parameters and the relation of the gene polymorphisms with tumour-related survival.

## MATERIALS AND METHODS

### Patients and study design

Fresh histologically normal tissue specimens of 79 patients (21 females and 58 males, mean age 66 years, range 35–91 years) who underwent resection for primary gastric adenocarcinoma at the department of Oncologic Surgery of the Leiden University Medical Center were collected prospectively, as described before (Janssen *et al*, 2002). Various clinico-pathological data were (re-)evaluated or collected from patient files by one gastroenterologist and one pathologist (Janssen *et al*, 2002). All carcinomas were classified according to the TNM classification (Hermanek and Sobin, 1992) and localisation as well as diameters of the tumours were registered. Microscopical histological parameters, including differentiation-grade, classification according to WHO, Borrmann and Laurén, as well as the presence of *Helicobacter pylori* (*Hp*) and intestinal metaplasia in the normal gastric mucosa were assessed. All patients entered the study at operation date and a patient's time experience ended in the event of death or, when still alive, at the common closing date. The minimal follow-up was 33 months with a decreasing overall survival according to TNM stage, that is, from TNM I (*n*=23), to TNM II (*n*=24), to TNM III (*n*=25), and to TNM IV (*n*=7). Genomic DNA was isolated using the salting out method (Miller *et al*, 1988). In addition, DNA was extracted from peripheral blood leucocytes of 169 healthy volunteers (38% male, median age 33 years (range 18–73 years), >95% Caucasian) as described before (van der Veek *et al*, 2005).

### Single-nucleotide polymorphism analyses

Genotypes were analysed by PCR-based techniques as described in Table 1.

### Antigen determination and protein concentration

From 50–100 mg of wet tissue samples, homogenates were prepared. The samples were wet weighted, and 1 ml of 0.1 M Tris-HCl (pH 7.5) with 0.1% (v.v^−1^) Tween-80 extraction buffer per 60 mg sample was added as described previously. The protein concentration was determined using the method of Lowry *et al* (1951). Specific ELISAs for the MMP and TIMP antigen determination were performed as recently described (Kubben *et al*, 2006).

### Statistical analysis

Statistical analyses were performed using SPSS11.0 Statistical Package (2004, SPSS Inc., Chicago, IL, USA). Hardy–Weinberg analysis was performed using the chi-square (*χ*^2^) or Fisher's exact test to examine differences in the distribution of alleles and genotypes between patients and controls. Odds ratios and confidence intervals (95%) were calculated by logistic regression. For the tumour-related survival analysis, the clinico-pathological parameters were dichotomised as described before (Sier *et al*, 1996). Univariate survival analyses were performed with the Cox proportional hazards model, using the clinico-pathological parameters and MMP-SNPs, resulting in the identification of covariates that significantly correlated with the survival of the patients. Multivariate survival analysis was performed by separately adding the MMP-SNPs variables to all the dichotomised clinico-pathological parameters. Tumour-related survival curves were constructed using the method of Kaplan and Meier including the log rank test. Group means for antigen levels were compared using two-tailed Mann–Whitney *U*-tests. Differences were considered significant when *P*⩽0.05.

## RESULTS

The genotype distribution and allele frequencies of the SNPs for MMP-2, -7, -8, -9, TIMP-1 and -2 for the 79 gastric cancer patients and 169 control subjects are summarised in Table 2. Single-nucleotide polymorphisms −1306C>T and −1575G>A for MMP-2 were found to be in complete linkage disequilibrium and consequently, in the rest of the study only MMP-2_−1306C>T_ will be described. None of the genotype distributions in the control group or in the cancer patients deviated from the Hardy–Weinberg equilibrium (data not shown). Matrix metalloproteinase-7_−181A>G_ was the only polymorphism differently distributed among gastric carcinoma patients compared with control subjects: AA 43.0%, AG 46.8%, and GG 10.1% in patients *vs* AA 27.2%, AG 62.7% and GG 10.1% in controls (*P*<0.04; Table 2). Comparison of the genotype distribution of our Caucasian control subjects with those published on other mainly Asiatic control groups (Wollmer *et al*, 2002; Ghilardi *et al*, 2003; Krex *et al*, 2003; Miao *et al*, 2003; Wang *et al*, 2004; Zhou *et al*, 2004; Matsumura *et al*, 2005; Zhang *et al*, 2005) showed significant differences for MMP-2_−1306C>T_, MMP-7_−181A>G_, TIMP-1_372C>T_ and TIMP-2_−418G>C_ (Table 3).

All the SNPs were evaluated for association with the clinico-pathological parameters. Correlations were found for MMP-2_−1306C>T_ with Borrmann's classification (fungating *vs* infiltrating: CC 70% and CT/TT 30% *vs* CC 48% and CT/TT 52%; *χ*^2^ 3.5, *P*=0.06), MMP-7_−181A>G_ with the presence of *Hp* (negative *vs* positive: AA 60% and AG/GG 40% *vs* AA 21% and AG/GG 79%; *χ*^2^ 7.8, *P*=0.005) and TIMP-2_303C>T_ with the WHO classification (differentiated *vs* not differentiated: CC 93% and CT/TT 7% *vs* CC 72 and CT/TT 28%; *χ*^2^ 5.9, *P*=0.03).

**Figure 1:**
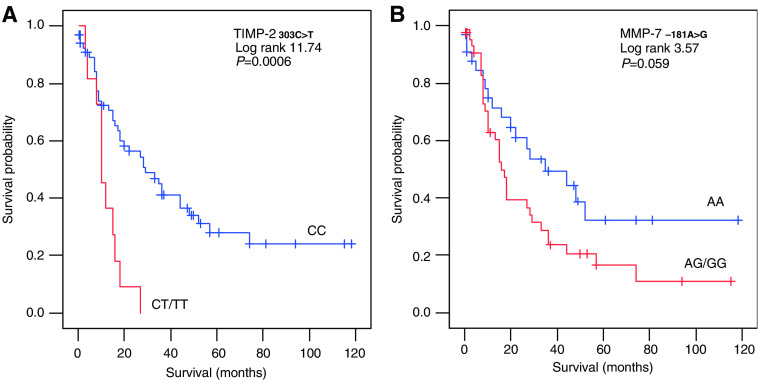
Survival curves using tumour-related death for 79 gastric cancer patients subdivided by the presence of a SNP in (**A**) the TIMP-2 gene (303C>T) and (**B**) the MMP-7 gene (−181A>G).

**Figure 2:**
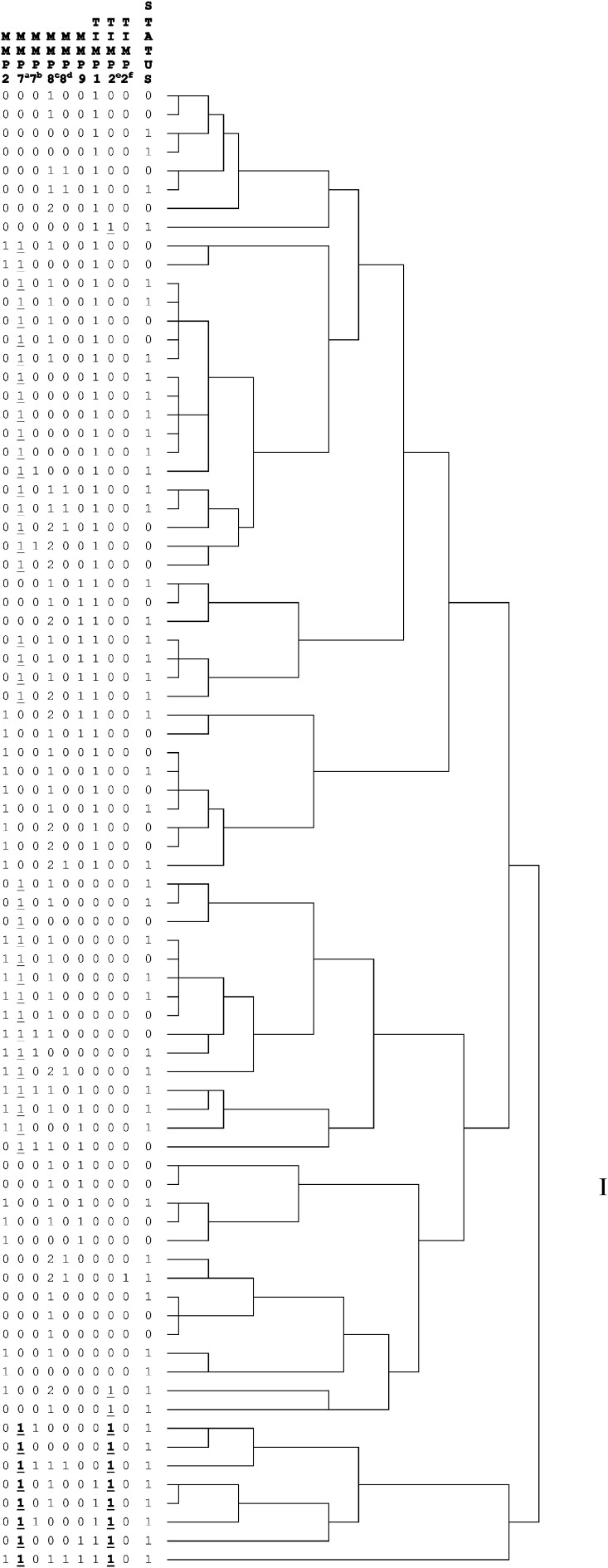
Dendrogram of a two-dimensional unsupervised hierarchical cluster analysis for 79 gastric cancer patients using SNPs of MMP-2_−1306C>T_, MMP-7_−181A>C_ (**A**), _−153C>G_ (**B**), MMP-8_−799C>T_(**C**), _+17C>G_(**D**), MMP-9_−1562C>T_, TIMP-1_372C>T_, and TIMP-2_303C>T_(**E**), _−418G>C_(**F**). For all the SNPs, 0 stands for the reference genotype and 1 for the combined other genotypes as described in Table 2. Because of the distribution, for MMP-8_−799C>T_(**C**) a three-group subdivision was used: 0=CC, 1=CT, 2=TT. Status: 0=alive or not tumour-related death, 1=tumour-related death.

**Figure 3:**
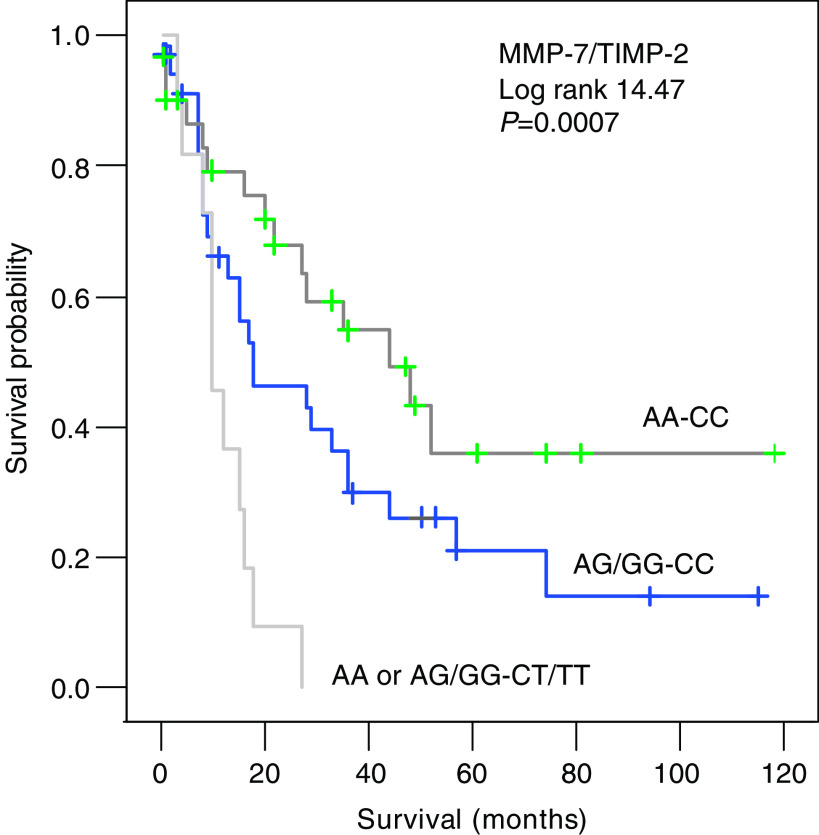
Survival curves using tumour-related death for 79 gastric cancer patients subdivided by the presence of combined polymorphisms in the MMP-7 gene (−181A>G) and TIMP-2 gene (303C>T).

The prognostic value for tumour-related survival of the respective SNPs was analysed using Cox proportional hazards analyses (Table 4). In the univariate analyses, TIMP-2_303C>T_ was significantly correlated with survival (Figure 1A), whereas MMP-7_−181A>G_ showed a trend (Figure 1B). From the clinico-pathological parameters, only TNM classification and the presence of intestinal metaplasia were significantly associated with survival, whereas the localisation showed a trend. In a multivariate analysis against all the clinical parameters TIMP-2_303C>T_ kept its significance, indicating its potential value as an independent prognostic marker. A dendrogram showing a two-dimensional unsupervised hierarchical cluster analysis for all 79 patients using all the SNPs determined in this study is presented in Figure 2. Interestingly, the first-order cluster (I) separated the eight patients with mutations in both the survival-associated SNPs, that is, MMP-7_−181A>G_ and TIMP-2_303C>T_, from the rest of the patients. Further analyses of this SNP combination revealed a stepwise and statistically significant poorer tumour-related survival for these mutations (0% (0 out of 11 patients) *vs* 32% (12 out of 37 patients) *vs* 52% (16 out of 31 patients); *χ*^2^ 9.7, *P*⩽0.01). Cox analyses confirmed this prognostic significance of this MMP-7_−181A>G_ – TIMP-2_303C>T_ combination, as indicated in Table 4 and illustrated in Figure 3.

The relation between the genotype distribution of the SNPs and the protein levels in normal and tumour tissue is shown in Table 5. As expected, the exon-located SNPs were not found to be accompanied by changes in the respective protein levels. The promoter-located SNPs showed some trends with the protein levels, but the only relevant significant difference was found for MMP-2_−1306C>T_ within tumour tissue.

## DISCUSSION

Because some gene polymorphisms of MMPs and TIMPs have been found to be related to disease susceptibility and changed gene transcription *in vitro*, we investigated whether gastric cancer is associated with SNPs of MMP-2, -7, -8 and -9, or their inhibitors TIMP-1 and TIMP-2. The only SNP that was distributed significantly differently among gastric carcinoma patients compared to our control population was MMP-7_−181A>G_, with more patients of the AA genotype than in controls. The latter was not expected from previous studies on gastrointestinal cancer (Ghilardi *et al*, 2003; Zhang *et al*, 2005) and is most likely caused by ethnic differences (Asiatic *vs* Caucasian; Table 3), disease localisation (gastric *vs* colon) and the relatively low number of patients included in the studies. In our study, the gastric cancer patients with the variant AG/GG genotype showed worse survival data than the AA patients (Table 4 and Figure 1B), although the difference did not fully reach statistical significance. The fact that tumours of the AG/GG patients did not contain higher MMP-7 antigen levels in our study suggests that the presence of SNP MMP-7_−181A>G_ alone is not directly translated into an enhanced tumour MMP-7 antigen expression or activity. However, considering the previously shown localised presence of MMP-7 at the invasive front of tumours, immunohistochemical or *in vitro* studies might further elucidate this functional relationship. The other striking correlation of MMP-7_−181A>G_ in this study is with the presence of *Hp*. Gastric cancer patients with the AG/GG genotype were significantly more often *Hp*-positive, which might indicate an enhanced susceptibility for this bacterium. The presence of *Hp* is associated with the development of gastric cancer and stimulation of MMP-7 production by *Hp* in human gastric epithelial cells has previously been suggested as a possible mechanism predisposing towards gastric neoplasia (Wroblewski *et al*, 2003; Chen *et al*, 2004).

Tissue inhibitor of metalloproteinase-2 is involved in the regulation of MMP-2 activity (Howard *et al*, 1991; Wang *et al*, 2000). In addition, TIMP-2 has been shown to promote cell growth (Hayakawa *et al*, 1994). Enhanced amounts of TIMP-2 protein are found to be associated with prostate cancer malignancies (Ross *et al*, 2003), but for colon and gastric cancer the correlation with clinico-pathological parameters has not been established (Ring *et al*, 1997; Joo *et al*, 2000). In our study, the CT/TT variant of TIMP-2_303C>T_ was observed more frequently in undifferentiated gastric carcinomas (WHO classification) and it was associated with worse tumour-related survival of gastric cancer patients. Tissue inhibitor of metalloproteinase-2_303C>T_ is located in exon 3 with no effect on the final amino-acid sequence of the protein (S101S) and no effect on the total TIMP-2 expression between gastric normal and tumour tissue (Table 5). Therefore, the TIMP-2_303C>T_ SNP behaves as a disease susceptibility gene polymorphism by a so far undefined mechanism. The other SNP for TIMP-2 in this study (−418G>C), localised in the promoter of the gene, has been described to abolish the Sp1-binding site and therefore may downregulate TIMP-2 gene expression (Hirano *et al*, 2001). A previous study reported that the variant TIMP-2_−418G>C_ genotype (GC or CC) was indeed associated with a moderately reduced risk of breast cancer in a Chinese population (Zhou *et al*, 2004). Because our group of Caucasian gastric cancer patients contained only one patient with the variant genotype (GC), we could not determine an association with tumour staging, patient survival or antigen expression.

The first-order cluster in a two-dimensional unsupervised hierarchical cluster analysis including all SNPs clearly separated the patients with mutations in both the survival-associated SNPs, that is, MMP-7_−181A>G_ and TIMP-2_303C>T_ from the rest of the patients. Cox analysis confirmed this SNP combination as a prognostic parameter for gastric cancer. Although results of cluster analysis of SNPs in gastric cancer have not been published before, hierarchical cluster analysis of patterns of chromosomal aberrations in gastric cancer patients identified patients with worse prognosis as well (Weiss *et al*, 2003), confirming the validity of such an approach.

The (−1306C>T) SNP in the promoter of the MMP-2 gene has also been found to diminish promoter activity by abolishing the Sp1-binding site (Price *et al*, 2001). Consequently, the variant genotypes (CT/TT) are expected to produce less MMP-2 antigen, which consequently might be associated with decreased cancer risk or better survival of the patients (Sier *et al*, 1996). Although we did not find a significant difference in distribution of MMP-2_−1306C>T_ between gastric cancer patients and controls, the tumours from patients with the CT/TT genotypes contained significantly less MMP-2 antigen than the CC genotype (Table 5). This relation was expected, but as far as we know, never shown before. The fact that the MMP-2_−1306C>T_ status on its own was not correlated with survival might be explained by the complicated activation mechanism of MMP-2 in which several other proteins are involved. Changes in MMP-2 antigen levels are therefore not directly correlated with MMP-2 activity levels. The fact that we did not find a relation with survival in our group of patients supports the study of Miao *et al* (2003), describing that the CC genotype was not associated with higher risk of metastasis at the time of diagnosis. A weak but significant difference in genotype distribution of MMP-2_−1306C>T_ and gastric carcinomas, classified according to the Borrmann classification, was observed with the highest percentage of the CC genotype in type 1/2 (fungating) preceding infiltrating tumours (type 3/4). This underscores the role of MMP-2 in breaking down the extracellular matrix in early gastric cancer which has been suggested before (Miao *et al*, 2003).

The genotype distribution of MMP-9_−1562C>T_ in our group of healthy controls was not different from other publications. We did not find differences in genotype distribution for MMP-9_−1562C>T_ between gastric cancer patients and controls either, which is in agreement with the study of Matsumura *et al* (2005) in Japanese patients. However, that study showed significant associations of the CT/TT genotype with depth of invasion, lymphatic invasion and TNM classification. In our study, MMP-9_−1562C>T_ was not correlated with clinico-pathological parameters or survival. Moreover, MMP-9 antigen levels in normal as well as tumour tissue of gastric cancer patients with the MMP-9_−1562C>T_ genotype were not enhanced, as was recently also found in plasma of healthy subjects (Demacq *et al*, 2006). Our results indicate that the presence of the T allele variant in the MMP-9 promoter (_−1562C>T_) is not associated with clinical outcome in our Caucasian group of gastric cancer patients.

Neutrophils secrete both gelatinase B (MMP-9) and neutrophil collagenase (MMP-8) after stimulation. Matrix metalloproteinase-8 expression levels correlated with tumour stage and poor prognosis in ovarian cancer (Stadlmann *et al*, 2003). Levels of MMP-8 and -9 correlated significantly with each other and with TIMP-1 levels, but were not related to tumour size or prognosis in human breast cancer (Duffy *et al*, 1995). Nothing has been published thus far about SNPs for MMP-8 and cancer, but three MMP-8 promoter haplotypes (MMP-8_–799C>T_, MMP-8_+17C>G_ and MMP-8_−381A>G_) have been found to be associated with preterm rupture of membranes in delivery, indicating a functional role on MMP-8 expression (Wang *et al*, 2004). Because MMP-8_+17C>G_ and MMP-8_−381A>G_ were found to be in complete linkage disequilibrium, we decided to study the distribution of MMP-8_–799C>T_, MMP-8_+17C>G_ in our group of gastric cancer patients. However, we did not find any relation of both SNPs with protein levels, clinico-pathological parameters, or survival in this study.

TIMP-1 is a ubiquitous glycoprotein capable of inhibiting all activated collagenases (Gomez *et al*, 1997). Tissue inhibitor of metalloproteinases were previously found not to be correlated with tumour stage, histological type, lymph node status or survival in human gastric cancer (Murray *et al*, 1998). We did not find any relation of TIMP-1_372C>T_ with gastric carcinoma, protein level or survival of the patients.

Taken together, our data indicate that MMP and TIMP gene polymorphisms contribute to gastric carcinogenesis. Determination of these gene polymorphisms, especially MMP-7_−181A>G_ and TIMP-2_303C>T_ both as single parameter and in combination as a cluster, might be helpful to identify gastric cancer patients with a poor clinical outcome and in need of (neo)-adjuvant treatment aiming at better outcome.

## Figures and Tables

**Table 1:** Primer sequences and PCR conditions for amplification of MMP and TIMP SNPs

**SNP**	**Method**	**Primer**	**Sequence**	**Location**	**Annealing**	**BP**	**Enzyme**	**Reference**
MMP-2_−1575G>A_	RFLP-PCR	Outer primers	ACCAGACAAGCCTGAACTTGTCTGA	Promoter	63°C, 35 cycles	542	*Bsp*HI	(Harendza *et al*, 2003)
			TGTGACAACCGTCTCTGAGGAATG					
MMP-2_−1306C>T_	Tetra-primer ARMS-PCR	Outer forward	ACCAGACAAGCCTGAACTTGTCTGA	Promoter	63°C, 35 cycles	542		(Ye *et al*, 2001)
		Outer reverse	TGTGACAACCGTCTCTGAGGAATG			3792		
		Inner forward	ATATTCCCCACCCAGCACGCT			11		
		Inner reverse	GCTGAGACCTGAAGAGCTAAAGAGTTG					
MMP-7_−181A>G_	RFLP-PCR	Forward	TGGTACCATAATGTCCTGAATG	Promoter	55°C, 35 cycles	150	*Eco*RI	(Jormsjö *et al*, 2001)
		Reverse mismatch	TCGTTATTGGCAGGAAGCACACAATGAATT					
MMP-7_−153C>G_	RFLP-PCR	Forward mismatch	ACGAATACATTGTGTGCTTCCTGCCAATCA	Promoter	55°C, 30 cycles	158	*Nla*III	(Jormsjö *et al*, 2001)
		Reverse	TTTATATAGCTTCTCAGCCTCG					
MMP-8_−799C>T_	RFLP-PCR	Forward	CTGTTGAAGGCCTAGAGCTGCTGCTCC	Promoter	58°C, 35 cycles	968	*Sfc*I	(Wang *et al*, 2004)
		Reverse	CATCTTCTCTTCAAACTCTACCC					
MMP-8_+17C>G_	RFLP-PCR	Forward	CTGTTGAAGGCCTAGAGCTGCTGCTCC	Transcription start	58°C, 35 cycles	668	*Dde*I	(Wang *et al*, 2004)
		Reverse	CATCTTCTCTTCAAACTCTACCC					
MMP-9_−1562C>T_	RFLP-PCR	Forward	ATGGCTCATGCCCGTAATC	Promoter	60°C, 38 cycles	352	*Nla*III or *Sph*I	(Zhang *et al*, 1999)
		Reverse	TCACCTTCTTCAAAGCCCTATT					
TIMP-1_372C>T_	RFLP-PCR	Forward	GCACATCACTACCTGCAGTC	Exon 5 phe 124 phe	54°C, 35 cycles	175	*Bss*SI	(Wollmer *et al*, 2002)
		Reverse	GAAACAAGCCCACGATTTAG					
								
TIMP-2_−418G>C_	RFLP-PCR	Forward	CGTCTCTTGTTGGCTGGTCA	Promotor	64°C, 35 cycles	304	*Bso*BI	(Zhou *et al*, 2004)
		Reverse	CCTTCAGCTCGACTCTGGAG					
TIMP-2_303C>T_	RFLP-PCR	Forward	TAGGAACAGCCCCACTTCTG	Exon 3 ser 101 ser	60°C, 35 cycles	119	*Tsp*RI	(Krex *et al*, 2003)
		Reverse	CCTCCTCGGCAGTGTGTG					

ARMS=amplification refractory mutation system; MMP=matrix metalloproteinase; PCR=polymerase chain reaction; RFLP=restriction fragment length polymorphism; SNP=single-nucleotide polymorphism; TIMP=tissue inhibitor of metalloproteinase.

Deliberate mismatches in primers are underlined.

**Table 2:** Allele frequencies and genotype distribution of MMP and TIMP SNPs in gastric carcinoma patients (*n*=79) and controls (*n*=169)

		**Patients**									**Controls**												
**SNP**			***n***	**%**		***n***	**%**		***n***	**%**		***n***	**%**		***n***	**%**		***n***	**%**	***χ* ^2^**	***P***	**OR**	**CI**
MMP-2_−1306C>T_	Allele	C	124	78.5				T	34	21.5	C	257	76.0				T	81	24.0	0.362	NS		
	Genotype	CC^*^	50	63.3	CT	24	30.4	TT	5	6.3	CC	102	60.4	CT	53	31.4	TT	14	8.3	0.361	NS	0.833	0.51–1.53
MMP-7_−181A>G_	Allele	A	105	66.5				G	53	33.5	A	198	58.6				G	140	41.4	2.810	NS		
	Genotype	AA^*^	34	43.0	AG	37	46.8	GG	8	10.1	AA	46	27.2	AG	106	62.7	GG	17	10.1	6.533	<0.04	0.495	0.28–0.87
MMP-7_−153C>T_	Allele	C	149	94.3				T	9	5.7	C	320	94.7				T	18	5.3	0.029	NS		
	Genotype	CC^*^	70	88.6	CT	9	11.4	TT	—	0	CC	151	89.3	CT	18	10.7	TT	—	0	0.031	NS	1.079	0.46–2.52
MMP-8_−799C>T_	Allele	C	84	53.2				T	74	46.8	C	191	56.5				T	147	43.5	0.487	NS		
	Genotype	CC^*^	19	24.1	CT	46	58.2	TT	14	17.7	CC	55	32.5	CT	81	48.0	TT	33	19.5	2.509	NS	1.524	0.83–2.80
MMP-8_+17C>G_	Allele	C	147	93.0				G	11	7.0	C	309	91.4				G	29	8.6	0.380	NS		
	Genotype	CC^*^	68	86.1	CG	11	13.9	GG	—	0	CC	141	83.4	CG	27	16.0	GG	1	0.6	0.660	NS	0.781	0.37–1.66
MMP-9_−1562C>T_	Allele	C	137	86.7				T	21	13.3	C	286	84.6				T	52	15.4	0.376	NS		
	Genotype	CC^*^	59	74.7	CT	19	24.0	TT	1	1.3	CC	120	71.0	CT	46	27.2	TT	3	1.8	0.394	NS	0.830	0.45–1.52
TIMP-1_372C>T_	Allele	C	74	46.8				T	84	53.2	C	167	49.4				T	171	50.6	0.285	NS		
	Genotype♀	CC^*^	5	23.8	CT	10	47.6	TT	6	28.6	CC	24	22.4	CT	59	55.2	TT	24	22.4	0.481	NS	0.925	0.31–2.79
	♂	C^*^	27	46.6				T	31	53.4	C	30	48.4				T	32	51.6	0.040	NS	1.076	0.53–2.21
TIMP-2_303C>T_	Allele	C	146	92.4				T	12	7.6	C	301	89.0				T	37	11.0	1.359	NS		
	Genotype	CC^*^	68	86.1	CT	10	12.7	TT	1	1.3	CC	133	78.7	CT	35	20.7	TT	1	0.6	2.588	NS	0.598	0.29–1.25
TIMP-2_−418G>C_	Allele	G	157	99.4				C	1	0.6	G	337	99.7				C	1	0.3	0.305	NS		
	Genotype	GG^*^	78	98.7	GC	1	1.3	CC	—	0	GG	168	99.4	GC	1	0.6	CC	—	0	0.306	NS	2.154	0.13–34.9

CI=confidence interval; MMP=matrix metalloproteinase; NS=not significant; OR=odds ratio; PCR=polymerase chain reaction; SNP=single-nucleotide polymorphism; TIMP=tissue inhibitor of metalloproteinase.

The *χ*^2^ test was used to examine differences in the distributions of alleles and genotypes between patients and controls.

OR and 95% CI were calculated by logistic regression using marked genotypes (^*^) as reference groups.

**Table 3:** Comparison of genotype distributions of the control subjects from this study (*n*=169, 107♀/62♂) with the control groups from previously published studies

**MMP-2 _−1306C>T_**	**MMP-7 _−181A>G_**	**MMP-7 _−153C>T_**	**MMP-8 _−799C>T_**	**MMP-8 _+17C>G_**	**MMP-9 _−1562C>T_**	**TIMP-1 _372C>T_**	**TIMP-2 _303C>T_**	**TIMP-2 _−418G>C_**
Lin *et al*, 2004	Ghilardi *et al*, 2003	Ghilardi *et al*, 2003	Wang *et al*, 2004	Wang *et al*, 2004	Demacq *et al*, 2006	Krex *et al*, 2003	Krex *et al*, 2003	Hirano *et al*, 2001
*n*=147 (A)	*n*=111 (C)	*n*=111 (C)	*n*=216 (B)	*n*=216 (B)	*n*=200 (♂C)	*n*=24♀/20♂ (C)	*n*=41 (C)	*n*=40 (A)
*χ*^2^ 6.0	*χ*^2^ 1.7	*χ*^2^ 1.7	*χ*^2^ 3.8*	*χ*^2^ 0.1*	*χ*^2^ 5.8	♀*χ*^2^ 4.1, ♂*χ*^2^ 5.0	*χ*^2^ 0.3	*χ*^2^ 66.6
NS	NS	NS	NS	NS	NS	NS, *P*⩽0.025	NS	*P*⩽0.001
								
Miao *et al*, 2003	Zhang *et al*, 2005				Lose *et al*, 2005	Lose *et al*, 2005	Wang *et al*, 1999	Zhou *et al*, 2004
*n*=789 (A)	*n*=350 (A)				*n*=392 (C)	*n*=34♀/33♂ (C)	*n*=82 (C)	*n*=509 (A)
*χ*^2^ 16.7	*χ*^2^ 217.2				*χ*^2^ 0.7	♀*χ*^2^ 8.2, ♂*χ*^2^ 1.3	*χ*^2^ 3.7*	*χ*^2^ 66.7
*P*⩽0.001	*P*⩽0.001				NS	NS, NS	NS	*P*⩽0.001
								
(Xu *et al*, 2004)					Matsumura *et al*, 2005	Wollmer *et al*, 2002		
*n*=126 (A)					*n*=224 (A)	*n*=159♀/114♂ (C)		
*χ*^2^ 8.6					*χ*^2^ 0.2	♀*χ*^2^ 8.0, ♂*χ*^2^ 0.0		
*P*⩽0.025					NS	*P*⩽0.025, NS		
								
Zhou *et al*, 2004								
*n*=509 (A)								
*χ*^2^ 23.1								
*P*⩽0.001								

MMP=matrix metalloproteinase; NS=not significant; TIMP=tissue inhibitor of metalloproteinase.

* Allele distribution. (A): Asiatic population, (B): Afro-American population, (C): Caucasian population.

**Table 4:** Univariate and multivariate Cox proportional hazard analysis for gastric cancer patients testing SNPs for MMP and TIMP *vs* clinico-pathological parameters

			**Univariate**			**Multivariate**		
**Parameter**		***n***	**HR**	**CI 95%**	***P***	**HR**	**CI 95%**	***P***
Gender	F *vs* M	21–58	0.706	0.390–1.278	NS	0.606	0.322–1.138	NS
Age	<median>	40–39	1.231	0.709–2.138	NS	1.422	0.749–2.701	NS
TNM	1	23	1	—	—	1	—	—
	*vs* 2	24	3.041	1.302–7.102	0.01	4.282	1.629–11.257	0.003
	*vs* 3	25	2.995	1.293–6.933	0.01	3.119	1.175– 8.280	0.022
	*vs* 4	7	7.175	2.420–21.271	0.0005	19.661	5.096–75.855	0.0005
Laurén	diffuse/mix *vs* intestinal	28–50	0.913	0.522–1.595	NS	1.281	0.344–4.774	NS
WHO	differentiated *vs* undiff.	53–25	1.152	0.652–2.033	NS	1.846	0.470–7.251	NS
Borrmann	fungating *vs* infiltrating	54–23	1.077	0.576–2.013	NS	0.677	0.338–1.356	NS
Localisation	Rest *vs* cardia	45–34	1.715	0.980–3.001	0.059	2.878	1.410–5.874	0.004
Diameter	⩽5 *vs* >5 cm	45–34	1.07	0.615–1.861	NS	0.612	0.324–1.158	NS
Intestinal metaplasia	Not *vs* present	37–42	0.499	0.283–0.880	0.016	0.704	0.378–1.312	NS
SNP								
MMP-2_−1306C>T_	CC *vs* CT/TT	50–29	0.756	0.421–1.358	NS	1.158	0.578–2.321	NS
MMP-7_−181A>G_	AA *vs* AG/GG	34–45	1.718	0.965–3.057	0.066	1.637	0.850–3.152	NS
MMP-7_−153C>T_	CC *vs* CT	70–9	1.096	0.467–2.575	NS	1.137	0.396–3.269	NS
MMP-8_−799C>T_	CC *vs* CT/TT	19–60	0.681	0.376–1.234	NS	0.607	0.302–1.222	NS
MMP-8_+17C>G_	CC *vs* CG	68–11	1.349	0.656–2.775	NS	1.364	0.516–3.606	NS
MMP-9_−1562C>T_	CC *vs* CT/TT	59–20	1.127	0.598–2.126	NS	1.006	0.482–2.101	NS
TIMP-1_372C>T_	CC *vs* CT/TT	32–47	1.125	0.644–1.967	NS	0.739	0.387–1.411	NS
TIMP-2_303C>T_	CC vs CT/TT	68–11	3.224	1.571–6.616	0.001	4.445	1.808–10.928	0.001
TIMP-2_−418G>C_	GG *vs* GC	78–1	ND	ND	ND	ND	ND	ND
MMP-7_−181A>G_ and	AA-CC	31	1	—	—	1	—	—
TIMP-2_303C>T_	*vs* AG/GG-CC	37	1.896	1.011–3.558	0.046	1.911	0.947–3.856	0.071
	*vs* AA or AG/GG-CT/TT	11	3.859	1.578–9.442	0.003	5.323	1.736–16322	0.003

CI=confidence interval; F=female; HR=hazard ratio; M=male; MMP=matrix metalloproteinase; ND=not defined; NS=not significant; SNP=single-nucleotide polymorphism; TIMP=tissue inhibitor of metalloproteinase; TNM=tumour node metastasis; WHO=World Health Organisation.

**Table 5:** Association between the presence of SNPs and the protein levels (mean±s.e.m. in ng mg^−1^ protein) within tissue of MMPs and TIMPs in 79 gastric carcinoma patients

**SNP**		**Protein level**					
**Located in promoter**		**Normal mucosa**		***P*-value**	**Tumour**		***P*-value**
MMP-2_−1306C>T_	CC *vs* CT/TT	5.0±0.5	4.5±0.7	NS	18.2±2.4	14.9±3.8	0.03
MMP-7_−153C>T_	CC *vs* CT^a^	2.2±0.6	0.7±0.0	0.019	47.1±14.1	46.1±16.4	NS
MMP-7_−181A>G_	AA *vs* AG/GG	1.3±0.4	2.1±0.6	NS	52.1±22.3	43.4±15.0	NS
MMP-8_−799C>T_	CC *vs* CT/TT	139±31	83±12	0.044	305±67	326±60	NS
MMP-8_+17C>G_	CC *vs* CG	98±19	95±15	NS	302±51	440±140	NS
MMP-9_−1562C>T_	CC *vs* CT/TT	9.7±1.1	7.0±1.5	NS	26.9±2.8	19.4±3.3	NS
TIMP-2_−418G>C_	GG *vs* GC^b^	6.0±0.3	5.1	NS	6.3±0.4	5.2	NS
Located in exon							
TIMP-1_372C>T_	CC *vs* CT/TT	8.7±1.6	7.7±0.7	NS	18.8±2.6	15.7±1.4	NS
TIMP-2_303C>T_	CC *vs* CT/TT	6.0±0.3	5.6±0.6	NS	6.0±0.4	7.5±1.6	NS

MMP=matrix metalloproteinase; NS=not significant; SNP=single-nucleotide polymorphism; TIMP=tissue inhibitor of metalloproteinase.

a *n*=3.

b *n*=1.
